# Temperature, Larval Diet, and Density Effects on Development Rate and Survival of *Aedes aegypti* (Diptera: Culicidae)

**DOI:** 10.1371/journal.pone.0087468

**Published:** 2014-02-03

**Authors:** Jannelle Couret, Ellen Dotson, Mark Q. Benedict

**Affiliations:** 1 Department of Biology, Emory University, Atlanta, Georgia, United States of America; 2 Division of Parasitic Diseases and Malaria, Center for Global Health, Centers for Disease Control and Prevention, Atlanta, Georgia, United States of America; 3 Dipartimento di Medicina Sperimentale e Scienze Biochimiche, Università di Perugia, Perugia, Italy; Universidade Federal do Rio de Janeiro, Brazil

## Abstract

Many environmental factors, biotic and abiotic interact to influence organismal development. Given the importance of *Aedes aegypti* as a vector of human pathogens including dengue and yellow fever, understanding the impact of environmental factors such as temperature, resource availability, and intraspecific competition during development is critical for population control purposes. Despite known associations between developmental traits and factors of diet and density, temperature has been considered the primary driver of development rate and survival. To determine the relative importance of these critical factors, wide gradients of conditions must be considered. We hypothesize that 1) diet and density, as well as temperature influence the variation in development rate and survival, 2) that these factors interact, and this interaction is also necessary to understand variation in developmental traits. Temperature, diet, density, and their two-way interactions are significant factors in explaining development rate variation of the larval stages of *Ae. aegypti* mosquitoes. These factors as well as two and three-way interactions are significantly associated with the development rate from hatch to emergence. Temperature, but not diet or density, significantly impacted juvenile mortality. Development time was heteroskedastic with the highest variation occurring at the extremes of diet and density conditions. All three factors significantly impacted survival curves of experimental larvae that died during development. Complex interactions may contribute to variation in development rate. To better predict variation in development rate and survival in *Ae. aegypti*, factors of resource availability and intraspecific density must be considered in addition, but never to the exclusion of temperature.

## Introduction

The rate of development and survival of organisms can vary greatly in response to many biotic and abiotic factors of the environment. Higher temperatures are often associated with faster development rate and have variable impacts on immature survival in insects [1]–[6]. Density-dependent competition in insects is also associated with delayed maturity [7]–[11] and increased juvenile mortality [12]–[16]. Similarly, food availability and nutrient quality have known associations with development rates and mortality [17]–[23]. Despite the demonstrated associations of diet and density with developmental life-history traits, temperature remains a primary focus to explain development rate variation in insects [3], [24]–[27]. Selection for shorter development times is strong relative to other life history traits [3], [28]. Thus, development time is an important candidate for understanding how a phenotype varies under different environmental conditions.

Differences observed in development times of the yellow fever mosquito *Aedes aegypti* Linnaeus from different geographical locations have been attributed to climatic differences [29]. Tun-Lin *et al.* [30] suggest local adaptation to temperature and other climatic variables is occurring as the mean development for *Ae. aegypti* populations from Raleigh, NC [4] and Israel [31] reared at the same temperatures differ by five days. Yet differences of up to 25 days are reported for a single population of *Ae. aegypti* in New South Wales at similar temperatures through experimental manipulation of diet and density during development [32]. Rather than being locally adapted, we expect that *Ae. aegypti* developmental phenotypes are highly plastic in response to several environmental factors. Empirical estimates of larval performance in response to gradients of three environmental factors in *Ae. aegypti* are rare with notable exceptions [32]–[34]. We seek to determine the relative importance of temperature, diet, and density on developmental performance as well as evaluate their interactive effects. Studying the relationship between environmental variables that impact mosquito biology is critical to guide public health controls and improve understanding of the epidemiology of vector-borne disease [35].

We estimate juvenile mortality and development rate of *Ae. aegypti* across four-level gradients of three factors: temperature, diet concentration, and intraspecific density. Determining the relative importance of multiple factors and evaluating potential interactions during development is foundational to understanding phenotypic responses of organisms to complex and changing environments [36]–[38]. Estimating the effects of environmental conditions on mosquito larvae is also critical information in the controlling of natural larval populations [39]. *Ae. aegypti*, once mature, vectors dengue virus, the most commonly transmitted arthropod-borne virus in the world (World Health Organization 2012), and yellow fever, one of the most lethal (World Health Organization, 2013).

## Methods

### *Ae. aegypti* colony

All experimental *Ae. aegypti* were reared from dried F2 eggs originating from wild caught eggs collected in Iquitos, Peru (Apperson, C., personal communication). Adults were supplied with 2% sugar solution at all times and offered a blood meal (human) five days after emergence. For maintenance of the population, one of the authors provided the blood meal with full consent (JC). In consultation with the Institutional Review Board (IRB) of CDC, the IRB process is aimed toward protecting research subjects and this action is not subject to review. Mosquito colonies were maintained at 28°C and 80% relative humidity with a 12L∶12D schedule and 30 minutes of gradual transition of light levels to simulate sunrise and sunset.

### Experiment

Larval development rate and survival were quantified in artificial containers over gradients of temperature, food concentration (mg/ml/day), and conspecific density. Performance across conditions was evaluated by measuring mortality rates during development (dead individuals/cup), time to pupation (days since hatching), and time to emergence (days since hatching). Dead larvae were removed daily to determine mortality and estimate survival curves. We estimated development time through the daily counting and removal of molts for each life stage from II-instar to adult emergence. The diet mixture used was comprised of beef-liver powder, tuna meal, and vitamin mix in water [40] and was tested at 1%, 2%, 4%, and 8% concentrations (10 mg/ml, 20 mg/ml, 40 mg/ml, and 80 mg/ml of diet mixture in deionized water respectively). Four initial density levels (10, 20, 40, and 80 larvae/cup) were tested. At initial densities, 500 µl of diet mixture was added to experimental cups. Each day the volume of the diet mixtures administered to containers was adjusted according to the daily number of larvae in that container in order to maintain a constant ratio of mg/larva/day (Table 1). This four by four array was tested across four temperatures (21°C, 24°C, 27°C, 30°C) resulting in 64 unique combinations. This three-factor and four level experimental design was replicated twice.

**Table 1:** Experimental design of diet, density, and resultant ratios of mg/larva/day.

Diet mixture	5 mg/500 µL	10 mg/500 µL	20 mg/500 µL	80 mg/500 µL
Initial density				
10 larvae	0.5	1	2	4
20 larvae	0.25	0.5	1	2
40 larvae	0.125	0.25	0.5	1
80 larvae	0.0625	0.125	0.25	0.5

Synchronous hatching was induced using a barometric chamber at 85 mmHg for 15 minutes. First-instar larvae were placed in water previously brought to the experimental treatment temperature. Larvae were left to mature for 12 h with 5 ml of a 2% w/v mixture of the larval diet in order to allow enough growth to facilitate the pipette transfer of the correct number of first-instar larvae into experimental containers. Following this period, first instar larvae were transferred to artificial rearing cups with 250 ml of filtered rainwater. Rearing containers were 473 ml white, plastic, cylindrical food containers (Bauman Paper Co., Lexington, KY). The volume of water in each cup was maintained at 250 ml throughout the experiment by adding water as needed to a fill-line marked in permanent marker. Temperature and relative humidity were logged each hour throughout the duration of the experiment and remained constant, maintained by environmental chambers at the Centers for Disease Control and Prevention insectary facilities (Atlanta, GA).

### Statistical analysis

All tests were computed using R v3.0.1 statistical programming language (R Development Core 2013). A general linear mixed effects model (GLMM) with Poisson error and log link with the nlme package v3.1-109 [41] was used to compare the dependent variables of mean duration of larval stages as well as the mean duration of the entire juvenile period from hatch to emergence (log_10_-transformed) to fixed variables of temperature, mg/ml/day of diet, initial density, and random factors of replicate, generation, and individual. Individual was included as a random factor due to the repeated measures of development time recorded daily for experimental containers. Development times were also averaged for each of the 64 treatments. These computed means represented independent observations characterizing each container allowing the use of a completely randomized (CR) ANOVA rather than a repeated/related measures ANOVA. The inverse of mean development time (1/hours of development) was used to both normalize (Shapiro-Wilk test) and linearize development with respect to temperature [42], [43]. Development rates of the larval stages as well as the development rate from hatch to emergence were computed for each treatment and analyzed using CR ANOVA. Mortality rate estimates were non-parametric and heteroskedastic and so were analyzed using the Kruskal-Wallis test. Survival functions were analyzed using Kaplan-Meier analysis with the survival package v2.37-4 [44] and the Weibull function. Mantel-Cox Log-Rank tests were performed to determine whether increases in temperature, diet, or density significantly affected survival.

## Results

### Development rate

Mean development time was not normal for larval stages (Shapiro-Wilk test, W = 0.7981, p<0.0001) or for the period from hatch to emergence (Shapiro-Wilk test, W = 0.8471, p<0.0001). The inverse of mean development time (1/hours of development) was used to both normalize (Shapiro-Wilk test, W = 0.977, p<0.2747; Shapiro-Wilk test, W = 0.9755, p<0.2312) and linearize development with respect to temperature [42], [43].

Mean development times from hatch to pupation (i.e. larval stages; Table 2) and from hatch to emergence (Table 3) were estimated across gradients of temperature, diet, and density. Development time of larval stages decreased at higher temperature across all diet and density treatments. Development time increased with increasing initial density level as well as decreasing diet level. The greatest variation in development time of larval stages occurred when the lowest diet level was paired with the higher initial density levels. The impact of diet level on development time from hatch to emergence was most evident at the highest initial density level.

**Table 2:** Mean development time of larval stages for all treatments with standard error in parentheses.

		Temperature °C			
		21	24	27	30
All diets and densities		12.71 (0.17)	10.68 (0.18)	9.36 (0.16)	8.62 (0.14)
Diet	1%	18.95 (0.47)	16.57 (0.53)	14.71 (0.4)	13.82 (0.42)
	2%	11.81 (0.19)	10.7 (0.2)	9.92 (0.20)	9.27 (0.16)
	4%	10.23 (0.11)	8.11 (0.11)	6.58 (0.94)	6.39 (0.08)
	8%	9.88 (0.09)	7.49 (0.05)	6.24 (0.12)	5.74 (0.06)
Density	80	14.35 (0.28)	12.61 (0.30)	11.32 (0.25)	10.26 (0.24)
	40	11.11 (0.17)	9.05 (0.17)	7.8 (0.17)	7.27 (0.12)
	20	10.2 (0.14)	7.88 (0.10)	6.54 (0.16)	6.00 (0.09)
	10	10.57 (0.14)	7.73 (0.09)	6.18 (0.12)	6.18 (0.11)

For each diet, values are averaged across density treatments. For each density, values are averaged across diet.

**Table 3:** Mean development time from hatch to emergence for all treatments with standard error in parentheses.

		Temperature °C			
		21	24	27	30
All diets and densities		16.23 (0.18)	13.16 (0.18)	11.51 (0.15)	9.92 (0.12)
Diet level	1%	22.62 (0.50)	19.19 (0.56)	16.58 (0.39)	14.51 (0.37)
	2%	15.34 (0.20)	13.26 (0.20)	11.88 (0.19)	10.87 (0.16)
	4%	13.63 (0.11)	10.55 (0.10)	9.14 (0.07)	8.07 (0.08)
	8%	13.37 (0.09)	9.98 (0.05)	8.45 (0.07)	7.18 (0.05)
Density	80	17.99 (0.30)	15.10 (0.31)	13.23 (0.25)	11.30 (0.22)
	40	14.48 (0.18)	11.62 (0.17)	10.21 (0.17)	8.95 (0.12)
	20	13.63 (0.13)	10.36 (0.13)	9.03 (0.10)	7.74 (0.10)
	10	14.14 (0.15)	10.18 (0.13)	8.70 (0.09)	7.82 (0.11)

For each diet, values are averaged across density treatments. For each density, values are averaged across diet.

**Figure 1:**
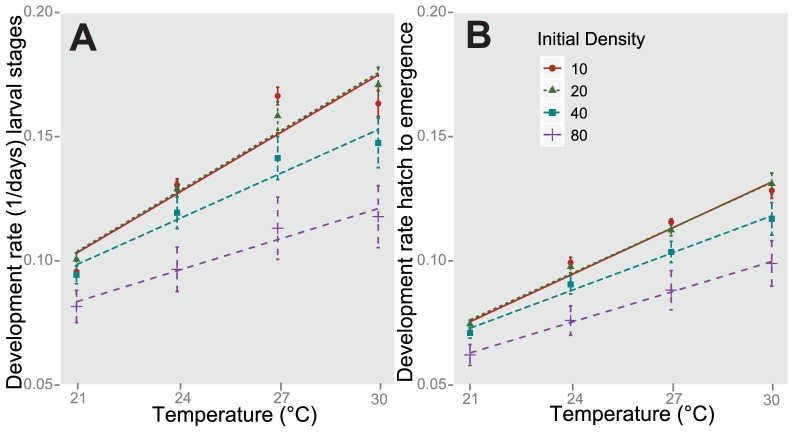
Mean development rate for larval stages (A) and from hatch to emergence (B). Bars indicate standard error. Character shape, color, and line type indicate initial density level. Lines indicate simple linear regression for density treatments.

Mean development rate (1/days of development time) of the larval stages was significantly impacted by all of the fixed independent factors and their two-way interactions (Table 4). Random factors of replicate, generation, and individual were not significant (Table 4). Initial densities of 40 and 80 larvae per cup had the greatest impact on reducing development rate (Fig. 1A). At the highest initial density (80 larvae/cup) the impact of mg/ml/day of diet was more evident with an average difference of 14.2 days between the lowest and highest diet levels across all four temperatures. In contrast, at the lowest initial density (10 larvae/cup) the average difference between the lowest and highest diets was 0.8 days across all temperatures. Similar results were seen for development time from hatch to emergence (Fig. 1B).

**Table 4:** CR ANOVA of temperature, diet (mg/ml/day), and initial density for development rate of larval stages.

	Df	SS	MS	F	p	
Temp	3	0.029	0.029	143.9	<0.000	***
Diet	3	0.021	0.021	104.4	<0.000	***
Density	3	0.018	0.018	88.19	<0.000	***
Temp×Diet	9	0.003	0.003	14.34	<0.000	***
Temp×Density	9	0.002	0.002	8.587	<0.001	**
Diet×Density	9	0.005	0.005	24.50	<0.000	***
Temp∶Diet∶Density	27	0.000025	0.000025	0.122	<0.8	

**Figure 2:**
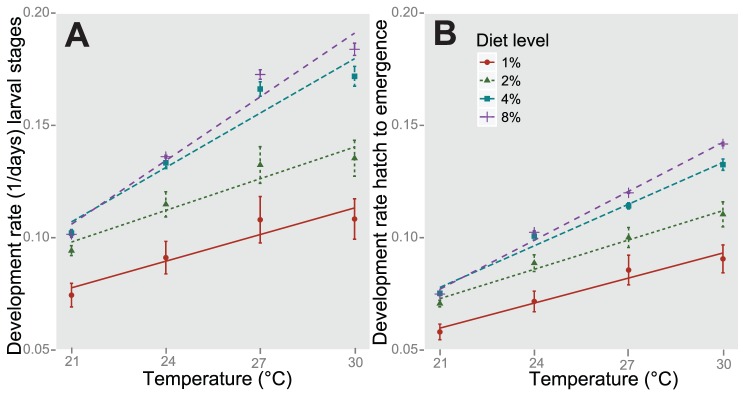
Mean development rate for larval stages (A) and from hatch to emergence across diet treatments. The amounts of each diet (mg/ml) were added to experimental cups daily. Bars indicate standard error. Character shape, color, and line type indicate diet treatment. Lines indicate simple linear regression for diet treatments.

Development rate of both the larval stages and the period from hatch to emergence was also significantly impacted by mg/ml/day of diet (Fig. 2). At the lowest diet level (0.02 mg/ml/day), the average difference between the lowest and highest density treatments was 14.4 days. At the highest diet level (0.16 mg/ml/day) the average difference is 0.4 days. The development rate for the larval stages was significantly associated at three of four temperature treatments (21°C, 24°C, and 27°C; F_2419,3_ = 295.392, p<2.2e-1). All three factors of temperature, diet, and density significantly explain the variation in mean development rate of larval stages (Table 4). These factors, as well as the two and three-way interactions, were significant for the development rate from hatch to emergence (Table 5).

**Table 5:** CR ANOVA of temperature, diet, and initial density for development rate from hatch to emergence.

	Df	SS	MS	F	p	
Temp	3	1.2152	0.4051	2424.8	<0.000	***
Diet	1	1.2481	1.2481	7471.4	<0.000	***
Density	1	0.3255	0.3255	1948.4	<0.000	***
Temp×Diet	3	0.1318	0.0439	263	<0.000	***
Temp×Density	3	0.0306	0.0102	61.1	<0.000	***
Diet×Density	1	0.1676	0.1676	1003.3	<0.000	***
Temp∶Diet∶Density	3	0.0115	0.0038	22.91	<0.000	***

**Figure 3:**
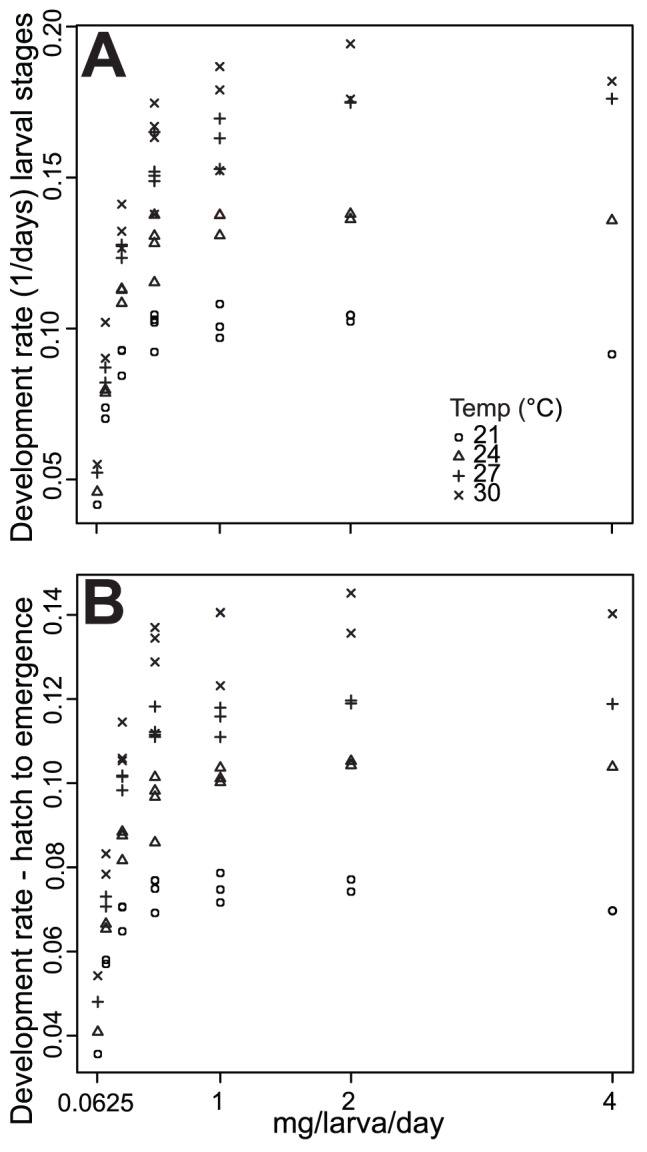
Development rate of larval stages (A) and from hatch to emergence (B) across mg/larva/day levels. Character shape represents temperature in which larvae were reared.

**Figure 4:**
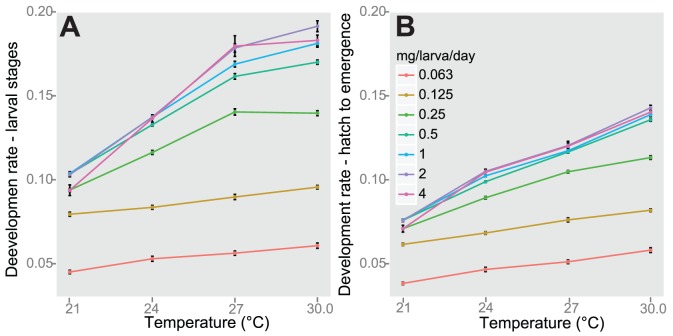
Development rate of larval stages (A) and hatch to emergence (B) across temperature and mg/larva/day. Line color indicates levels of mg/larva/day. Black bars indicate standard error.

We examined the changes in development rate with respect to the ratio of diet to density in order to assess the importance of the unit of mg/larva/day. By statistically comparing mg/larva/day within and between levels, we determined that the development rate of larval stages was not affected by higher initial numbers of larvae as long as the amount of mg/larva/day remained constant (Table 6). In comparing development of larval stages to mg/larva/day it was evident that as the amount of food per larva decreased, the differences between temperature treatments were smaller (Fig. 3A). In addition, as the amount of food per larva increased up to 1 mg/larva/day, the temperature treatment difference became apparent, but remained relatively constant at higher food doses (Fig. 3A). The same pattern was observed for the development rate from hatch to emergence (Fig. 3B). The relationship between temperature and larval development rate was significantly and positively linearly associated at each level of food/larva/day as determined by simple linear regression (Fig. 4A; Table S1). At 21°C, the lowest temperature treatment, there were smaller differences in development rate and differences increased with temperature such that the widest differences in development rate occurred at the 30°C (Fig. 4A). The same pattern was found with the development rate from hatch to emergence (Fig. 4B).

**Table 6:** Mean duration of larval stages for mg/larva/day across density levels and **χ^2^** tests.

		Initial density			?^2^	p
Ratio	10	20	40	80		
0.0625	*	*	*	20.89		
0.125	*	*	12.12	12.26	0.0008	0.98
0.25	*	9.22	8.34	8.5	0.0392	0.98
0.5	8.45	7.66	7.15	7.54	0.1162	0.99
1	7.82	6.92	7.15	*	0.063	0.97
2	7.21	6.84	*	*	0.0095	0.92
4	7.41	*	*	*		

Duration of larval stages reported in days. Ratios only possible at certain combinations of diet and density levels considered (see Table 1) otherwise indicated as *. Within ratio comparisons (Wald chi-square) were tested when two or more containers shared the same ratio of mg/larva.

### Survival

**Figure 5:**
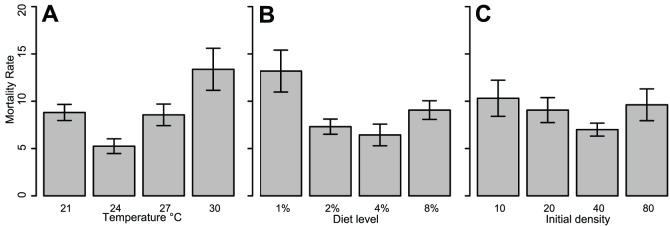
Mortality rate across temperature (A), diet concentration (B), and initial density (C).

**Figure 6:**
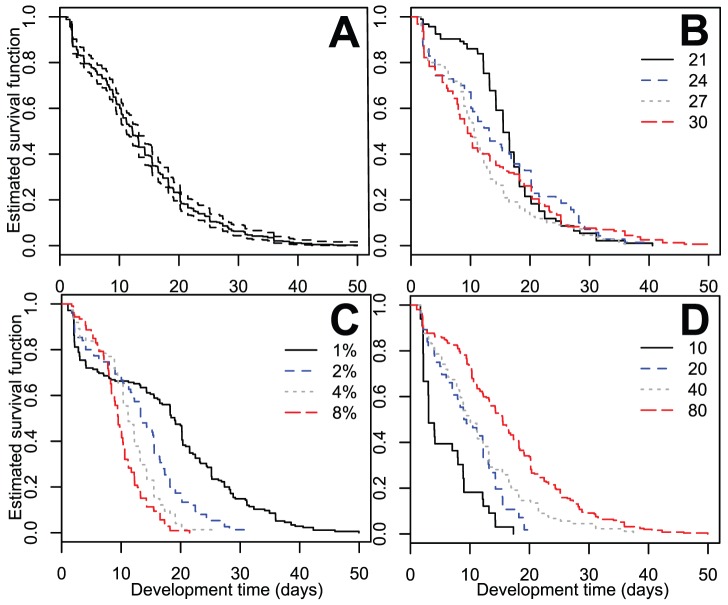
Survival curves over all treatments (A), by temperature (B), diet (C), and initial density (D). Treatments are distinguished by line type and color.

Mortality was defined as death during immature stages or during molting to the adult form. Of the 4,800 experimental larvae, 429 died for an overall mortality rate of 9% across all treatments (Fig. 5A–C). Mortality rate differed significantly across temperature treatments (Kruskal-Wallis, X^2^_df = 3_ = 10.79, p<0.05), but not diet (Kruskal-Wallis, X^2^_df = 3_ = 4.66, p>0.1) or initial density level (Kruskal-Wallis, X^2^_df = 3_ = 0.56, p>0.9). Survival curves were estimated based on the subset of larvae that died before or during emergence (n = 429; Fig. 6A). The larvae that survived to adulthood (n = 4,371) were excluded from analysis because their inclusion flattened the survival functions such that differences could not be visualized. Instead we examine the patterns of survival among those larvae that died before maturation. Temperature groups had significantly different survival functions (logrank, Mantel-Cox X^2^_df = 3_ = 8.39, p<0.05), and this impact appeared to be mainly driven by the 21°C treatment (Fig. 6B). Both diet (logrank, Mantel-Cox X^2^_df = 3_ = 105.4, p<0.0001) and initial density (logrank, Mantel-Cox X^2^_df = 3_ = 93.99, p<0.0001) demonstrated highly significant differences in survival functions, and these differences were evident at every level of each factor (Fig. 6C; Fig. 6D). While percent mortality was highest at the lowest diet level, larvae in these treatments also survived longer than those from higher diets. Larvae in higher density treatments survived longer than those in lower density treatments, and with similar mortality across densities (Fig. 6C).

## Discussion

We sought to estimate larval development and survival of *Ae. aegypti* across wide gradients of environmental conditions of temperature, diet, and density, and hypothesized all three factors and their interactions would influence variation in developmental timing and survival. During larval stages of mosquitoes, the aquatic environment can be experimentally manipulated to facilitate the study of environmental impacts on phenotypic variation both in the laboratory and under natural conditions [12], [19], [30], [45]–[49].

A GLMM model including factors of temperature, diet, and initial density and their two-way interactions best explains development rate variation for the larval stages (Table 3). For the entire developmental period from hatch to emergence, these factors as well as their two-way and three-way interactions are significant (Table 4). It is unclear why the developmental period from hatch to emergence would include a three-way interaction whereas the larval stages alone would not. The difference between these dependent variables is the exclusion or inclusion of metamorphosis, a complex developmental process to which plasticity in both juvenile and adult phenotypes has often been attributed [50]–[55]. Development rate differences in response to temperature have been observed between larval and pupal stages in the fruitfly *Drosophila buzzatii* [50]. Thus the impact and interaction of environmental factors may be stage-specific.

To progress to the next life stage, mosquito larvae require a minimum amount of nutrition in order to trigger hormonal developmental cascades [56]. The interactions observed here suggest that these thresholds of resource requirements are both temperature-dependent and density-dependent. Mechanisms for temperature-dependence of development rate include the impact of temperature on the use of fat body energy reserves [1] as well as the temperature-dependent growth of food resources of periphyton populations on which larvae feed [7]. The interactive effect of diet and density can be difficult to distinguish experimentally, and we chose an experimental design well-suited to this purpose [12], [57], [58].

Several hypotheses address the impact of crowding on mosquito larvae development including tactile interference [13], [34], [59], [60], chemical waste toxins, chemical signals exhibited by larvae, and stress from food partitioning (lower food per capita) at higher numbers. Ammonia accumulates in aquatic larval habitats due to larval waste and may retard [33] or accelerate [61], [62] growth. This may occur because ammonia influences the microbe populations on which mosquito larvae feed, or it may be a stressor to developing larvae. Growth retardant factor is also produced by overcrowded larvae, an effect that is not species-specific [33]. Growth retardant factor has been shown to lengthen development and prevent pupation in some Culicine mosquito species [63] but not others [13]. In a partitioned container designed to test the impact of shared water without the mechanical interference of crowding, Yoshioka et al. 2012 [45] do not find conspecific density to impact development time from hatch to emergence. These mechanisms are not mutually exclusive [13]. Our design does not distinguish between these factors, but rather focuses on distinguishing the impact of density from those of diet level, especially in the context of a limited resource environment. Our results indicate that larvae receiving the same amount of food per capita exhibit lower development rates at higher densities, an effect that is consistent across temperature.

At the lowest temperature, there were narrower differences between diet and density treatments than at intermediate and high temperatures (Fig. 3). The interaction between temperature, diet, and density may provide an alternative explanation of long-standing puzzle in insect physiology that insects are bigger when reared at colder temperatures. Many hypotheses focus on the impact of temperature to explain variation of life-history traits such as body size [64]–[66] and development rate [67]–[69].

Our results confirm a high degree of plasticity in development times in *Ae. aegypti* of a single brood that have as broad a range as distinct populations across continents and latitudes [18], [32], [70]–[79]. We cannot establish based on these data whether this plasticity is adaptive or non-adaptive. There is limited evidence suggesting that developmental timing and body size in mosquito larvae adaptively respond to changes in water volume [80]. Another potential mechanism for plasticity of developmental timing is adaptive plasticity of behaviors in mosquitoes. Behavioral changes in foraging in response to controphic competition [81] and oviposition [45] in response to conspecific larval density have been observed in mosquitoes.

While reduced larval survival with higher larval density has been observed in mosquito rearing studies [30], we did not find initial density to impact percent mortality of *Ae. aegypti*. This may be some indication that the initial density conditions here considered did not span a wide enough gradient to have a significant effect. In contrast, the schedule of survivorship among those larvae that died before emergence was significantly impacted by density as well as diet. Those larvae reared in lower diet concentrations survived longer than higher diet concentrations. This shift may be either a direct result of starvation on critical hormonal signals necessary for maturation [56] or another mechanism such as an increase in haemolymph lipid concentration that has been observed in insects under nutritional stress [82], [83]. In other Aedine mosquitoes, temperature has demonstrated effects on larval [2], [30] and adult [84] survival. Survival analysis in related species, *Aedes albopictus*, found no impact of either diet or density factors on the timing of survival over similar treatment levels as used in this study [45]. There is some evidence in other mosquito genera that temperature and density may interact to influence larval survival [85]. It may be that due to the same interactions observed for development rate, it is only when considering temperature, diet, and density that the impacts on survivorship curves become evident.

## Conclusions

Examining the plastic responses of *Ae. aegypti* to heterogeneous environmental conditions addresses broad questions in ecology and evolution [18], [19], [86], [87] as well as targeted public health questions [88]–[90]. Mosquitoes are historically important in the field of medical entomology, as vectors of human pathogens. In *Ae. aegypti*, recent evidence shows that shifting climatic patterns have impacted the timing of developmental stages [91] of this mosquito. There is an awareness that variation in environmental conditions and their impact on mosquito physiology [92]–[94] can influence vectorial capacity for dengue virus transmission [95], [96]. Even recent population dynamics models of *Ae. aegypti* and other mosquito vectors simplify the impact of environmental conditions to include only the influence of temperature [24], [97], [98].. Our results provide empirical estimates of life-history traits critical to modeling mosquito population abundance over wide gradients of these environmental conditions and illustrate the importance of interactive effects in modulating developmental timing. These findings support the need to include more complexity when predicting the population dynamics of this arboviral vector.

## Supporting Information

Table S1 **Parameter estimates and F tests of linear model of larval development rate from hatch to emergence and temperature, as shown in** **Figure 4** **.**
(DOCX)
