# Supplementary material for: Temperature, Larval Diet, and Density Effects on Development Rate and Survival of Aedes aegypti (Diptera: Culicidae)
Source: PLoS One. 2014 Feb 3;9(2):e87468. doi: 10.1371/journal.pone.0087468 (PMC3911954; doi:10.1371/journal.pone.0087468)
Supplement: Table S1 — Parameter estimates and F tests of linear model of larval development rate from hatch to emergence and temperature, as shown in Figure 4 . (DOCX) [file pone.0087468.s001.docx]

**Table S1.** Parameter estimates and F tests of larval development rate and temperature, as shown in Figure 4.

| **mg/larva/day** | **Slope** | **Intercept** | **Adj. R** | **F** | **p** |
| --- | --- | --- | --- | --- | --- |
| 0.0625 | 0.00155 | 0.00917 | 0.97 | 98.36 | 0.01 |
| 0.125 | 0.02593 | 0.01685 | 0.83 | 34.32 | 0.001 |
| 0.25 | 0.00483 | -0.00796 | 0.83 | 34.32 | 0.001 |
| 0.5 | 0.00689 | -0.04007 | 0.81 | 67 | 0 |
| 1 | 0.00796 | -0.06018 | 0.86 | 66.71 | 0 |
| 2 | 0.00944 | -0.09058 | 0.93 | 87.8 | 0 |
| 4 | 0.010387 | -0.118531 | 0.88 | 23.24 | 0.04 |
